# Experimental Study of Flame Dynamics in a Triple-Injector Swirling Nonpremixed Combustor Under Different Thermoacoustic Self-Excited Instability Modes

**DOI:** 10.3390/s25030850

**Published:** 2025-01-30

**Authors:** Xiang Zhang, Suofang Wang, Yong Liu

**Affiliations:** College of Energy and Power Engineering, Nanjing University of Aeronautics and Astronautics, Nanjing 210016, China; zhangxiang0321@nuaa.edu.cn (X.Z.); sfwang@nuaa.edu.cn (S.W.)

**Keywords:** thermoacoustic stability, flame dynamics, instabilities modes, combustion diagnosis, swirling nonpremixed flames

## Abstract

Combustion instability is one of the prominent and unavoidable problems in the design of high-performance propulsion systems. This study investigates the heat release rate (HRR) responses in a triple-nozzle swirling nonpremixed combustor under various thermoacoustic self-excited instability modes. Dynamic pressure sensors and high-speed imaging were employed to capture the pressure oscillations within the combustion chamber and the characteristics of flame dynamics, respectively. The results reveal nonlinear bifurcations in the self-excited thermoacoustic instabilities at different equivalence ratios. Significant differences in flame dynamics were observed across the instability modes. In lower frequency modes, the fluctuations in flame length contribute to the driving force of thermoacoustic instability. In relatively high-frequency modes, HRR fluctuations are dominated by the rolling up and convective processes of wrinkles on the flame surface. Alternating regions of gain and damping are observed on the flame surface. At even higher frequencies, both aforementioned HRR fluctuation patterns are simultaneously observed. These findings provide a deeper understanding of the complex interactions between flame dynamics and thermoacoustic instabilities, offering new insights into the design and optimization of nonpremixed combustion systems. The study underscores the importance of considering the spatial and temporal variations in flame behavior to effectively predict and control thermoacoustic instabilities.

## 1. Introduction

Thermoacoustic self-excited instability is a strong coupling phenomenon between acoustic pressure and heat release rate oscillations within combustion chambers. This coupling can lead to powerful pressure oscillations that are capable of damaging propulsion systems [1]. Despite extensive research spanning decades, thermoacoustic stability continues to pose a significant challenge in the development of modern high-performance propulsion systems [2,3]. This issue is particularly relevant to various types of engines, including liquid rocket engines [4], aircraft engines [5,6], and gas turbines [7,8].

Combustion instability is fundamentally a strong coupling between unsteady heat release and sound wave propagation within the combustion chamber, as famously described by Rayleigh’s criterion [1,9]. In recent years, numerous scholars have investigated the relationship between thermoacoustic instability and flame heat release response. Ann et al. [10] examined the differences in flame dynamics under identical intake velocities but different power outputs and hydrogen contents based on high-speed flame imaging results. They explained the mechanism of pressure oscillation amplitude under various modes using the spatial distribution of HRR pulsation. Kim et al. [11,12] employed experimental methods to study the HRR response of stratified swirl-stabilized premixed flames when there were significant velocity fluctuations at the combustor inlet. Han et al. [13] focused on the Beihang axial swirler independently stratified (BASIS) configuration, examining how the shape of the swirler outlet affects the spatial distribution of HRRs and combustion stability. Wang et al. [14,15,16] explored the relationship between flame dynamics characteristics and stability under fully premixed and partially premixed modes for the same combustor configuration. Specifically, partially premixed flames of value grade could broaden the range of flame thermoacoustic stability, whereas this phenomenon was not observed for main-grade, partially premixed flames. Under different thermoacoustic modes, the flames exhibited distinct structures. The authors pointed out that the thermoacoustic instability of different flame structures is primarily related to the coupled or decoupled modes of the thermoacoustic system. Fu et al. [17] concentrated on the differences in thermoacoustic stability and flame images under varying powers and equivalence ratios, as well as the impact of these parameters on pollutant emissions. Sanghyeok et al. [18] studied the influence of hydrogen content on the HRR response characteristics of two adjacent premixed flames, demonstrating that changes in H2 content significantly affect flame structure and subsequently influence the flame’s response characteristics to inlet velocity disturbances.

Recent studies on thermoacoustic stability have predominantly focused on swirling premixed flames. In contrast, investigations into the dynamic characteristics of nonpremixed flames in terms of thermoacoustic stability are relatively scarce. Liu et al. [19,20] analyzed the effects of different operating parameters on flame pulsation characteristics through flame image processing. Ahn et al. [21,22] examined the differences in nonlinear responses between nonpremixed and premixed flames, as well as flame separation under strong velocity disturbances, using simple Burke-Schumann flames as a model. Cao et al. [23] investigated oscillations in flame area, length, and expansion angle in the context of liquid rocket engines.

These research findings indicate that the pulsation characteristics of the HRR, as an important component of the thermoacoustic coupling feedback loop, often play a decisive role. In practical engineering applications, nozzles typically do not operate independently, such as those found in gas turbine combustors [7,8] and aircraft engine combustors [24,25]. However, there has been less focus on the role of sensor technologies and their application in capturing these complex dynamics.

In this study, dynamic pressure sensors and high-speed imaging techniques are employed to capture pressure oscillations within the combustion chamber and the characteristics of flame dynamics, respectively. By utilizing high-precision microphones to measure pressure fluctuations and photomultiplier tubes (PMTs) equipped with specific filters along with high-speed cameras to record HRR fluctuations, we were able to obtain detailed spatiotemporal resolution data. These sensors not only provide critical information about changes in flame morphology but also reveal unique characteristics of heat release rate fluctuations under different thermoacoustic instability modes. Specifically, through the analysis of time variations in OH* and CH* chemiluminescence intensity, we can more accurately quantify the heat release region, which is crucial for understanding the dynamic behavior of nonpremixed flames under varying equivalence ratio conditions.

This paper particularly focuses on triple-nozzle tangential swirl nonpremixed flames, investigating the impact of different equivalence ratio conditions on thermoacoustic stability under normal temperature and pressure environments. We will discuss in detail the differences in temporal and spatial pulsations of HRR under different instability modes. Through an in-depth analysis of sensor data, our research not only reveals the complex interactions between flame dynamics and thermoacoustic instabilities but also provides new insights for the design and optimization of nonpremixed combustion systems. Furthermore, this study underscores the importance of considering the spatial and temporal variations in flame behavior to effectively predict and control thermoacoustic instabilities. The paper is divided into four sections: Introduction (Section 1), Experimental Methods and Diagnostics (Section 2), Results and Discussion (Section 3), and Conclusions (Section 4).

## 2. Experimental Setup

### 2.1. Combustion Rig

**Figure 1:**
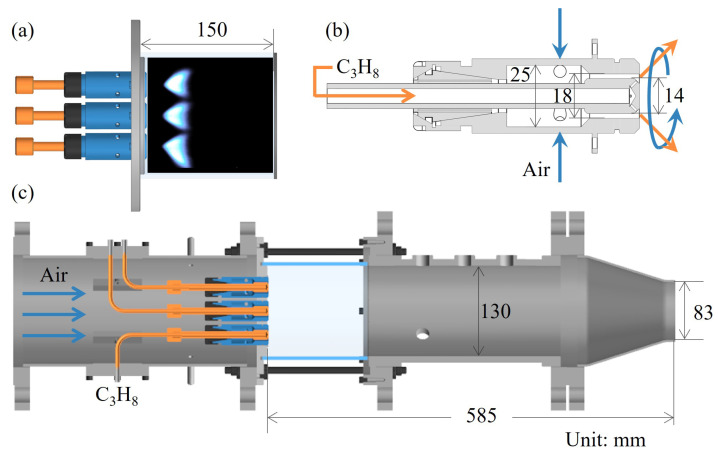
Schematic of the test rig: (**a**) the diagram of the triple-injector layout; (**b**) the structure of a single swirler injector; (**c**) the diagram of the model combustor.

This paper focuses on a combustion chamber equipped with three tangentially swirl-stabilized nonpremixed flame injectors, investigating the impact of operational parameters on thermoacoustic stability and flame dynamics characteristics. The layout of the combustion chamber is illustrated in Figure 1.

The three injectors are evenly spaced, as shown in Figure 1a. The spacing between the nozzles is designed to be 12 mm, with a center-to-center distance of 42 mm between adjacent nozzles.

Figure 1b provides a detailed structure of a single tangential swirl diffusion flame nozzle. The injector is centrally designed, with air entering the swirl chamber through four tangential inlets, each 5 mm in diameter, arranged at 90° azimuthal intervals. The airflow rotates clockwise relative to the flow direction. The swirl chamber has a diameter of 25 mm, and after passing through a contraction section, air enters the combustion chamber through the injector’s outer channel, which measures 18 mm in diameter. The central duct of the injector delivers fuel (C_3_H_8_), with the fuel injector having a diameter of 14 mm. Eight orifices, each 1 mm in diameter, are positioned at the fuel injector outlet, inclined at 45° to the chamber’s central axis. The swirl number (Sn) [26], a dimensionless parameter indicating the air swirl intensity, is defined as

(1) $$Sn=\frac{2G_{m}}{D_{\mathrm{sw}}G_{t}},$$

where $G_{m}$ is the tangential momentum flux, $G_{t}$ is the axial momentum flux at the swirl chamber exit, and $D_{\mathrm{sw}}$ is the exit diameter. $G_{m}$ and $G_{t}$ are calculated as [27]

(2) $$\begin{array}{ccc} G_{m} & =\int_{0}^{D_{\mathrm{sw}}/2}2\pi rWr\rho U,dr,\;G_{t} & =\int_{0}^{D_{\mathrm{sw}}/2}2\pi r\rho U^{2},dr. \end{array}$$

Here, *W* and *U* represent the tangential and axial velocity components, respectively. The injector flow was numerically simulated using the Reynolds-Averaged Navier-Stokes (RANS) method [28], with statistical calculations yielding an Sn of 0.96 at the injector outlet, indicative of a strong recirculation zone downstream, which is conducive to flame stabilization. The fuel mixes with the swirling air through the orifices and combusts.

The combustion chamber, with a diameter of 130 mm, features a contraction section and an outlet diameter of 83 mm. It measures 598 mm in total length, comprising a constant diameter section of 450 mm. A quartz observation window, 150 mm long, is installed in the upstream part of the combustion chamber for collecting the global heat release rate (HRR) and flame images during thermoacoustic instability. Three pressure sensor installation points, labeled P1 to P3, are spaced 60 mm apart along the chamber wall.

### 2.2. Diagnostic Techniques

**Figure 2:**
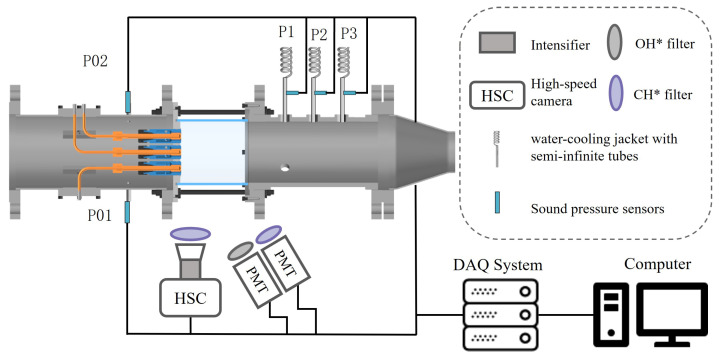
Schematic of the whole system.

The schematic of the whole system is shown in Figure 2. The combustion chamber is supplied with air from a compressor, regulated by a variable frequency drive, while C_3_H_8_ is sourced from a high-pressure fuel gas cylinder. Mass flow controllers (MFCs), specifically the AST10-H model with a capacity of 10 SLPM and an uncertainty of ≤1% FS, manage the flow rates of both air and fuel. A thermocouple measures the gas temperature at the combustion chamber outlet.

Pressure fluctuations during thermoacoustic instability are captured using a BSWA MPA471S microphone, characterized by a sensitivity of 0.9 mV/Pa, a dynamic range of 170 dB, and an uncertainty of ≤1% FS. These pressure measurements are positioned at 225 mm (P1), 285 mm (P2), and 345 mm (P3) from the injector outlet plane. Semi-infinite tubes are employed for all microphones to detect pressure oscillations. The tube section near the combustion chamber consists of a straight metal tube enclosed by a water-cooling jacket, which maintains a continuous flow of cooling water to keep the sensor within its operational temperature range. The tube’s other end connects to a 20 m closed tube to prevent wave reflections. A KELLER M5HB absolute pressure sensor, with a sensitivity of 3.3 V/bar, a range of 3 bar, and an uncertainty of ≤1% FS, measures pressure fluctuations in the fuel line. Flame HRR fluctuations are quantified via the combined intensity of OH and CH chemiluminescence [29]. The photomultiplier tube (PMT) for OH detection uses a 310 ± 10 nm filter, while another PMT for CH detection employs a 435 ± 5 nm filter. The HAMAMATSU CH253 PMT model, with a sensitivity of 250 μA/lm and an uncertainty of ≤1% FS, is utilized.

The spatial distribution of flame HRR during instability is documented by capturing CH luminescence images with a high-speed camera. A PHANTOM VEO 1010 high-speed camera, fitted with a 435 ± 5 nm narrowband filter, was used for this purpose. To enhance the luminosity of the CH chemiluminescence images, a Photonis Cricket image intensifier amplifies the CH signal. The camera is set to an exposure time of 50 μs and a frame rate of 5 kHz. All signals were collected using the NI PXle-1092 multichannel data acquisition (DAQ) system. The sampling frequency was set to 20 kHz.

**Figure 3:**
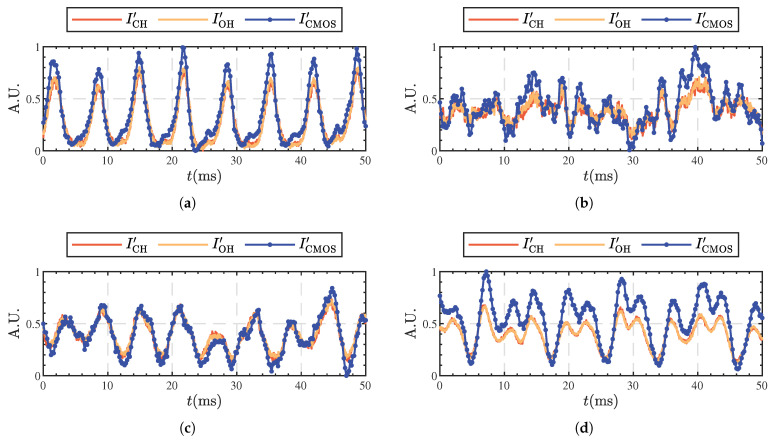
Time variations of normalized heat release rate signals: ϕ=0.212 (**a**), ϕ=0.270 (**b**), ϕ=0.414 (**c**), and ϕ=0.482 (**d**).

While CH chemiluminescence is a well-established indicator of the heat release region in premixed flame thermoacoustic instability studies [15,16], its application in hydrocarbon nonpremixed flames is less common. Thus, the integrated brightness of camera images was compared with PMT measurements, as depicted in Figure 3.

Under various equivalence ratios, the normalized global CH chemiluminescence intensity ($I_{\mathrm{CH}}^{\prime }$), global OH chemiluminescence intensity ($I_{\mathrm{OH}}^{\prime }$), and spatially integrated CH chemiluminescence images ($I_{\mathrm{CMOS}}^{\prime }$) show consistent phase and similar amplitudes at different times. This suggests that CH chemiluminescence images can accurately represent the spatial distribution and fluctuation characteristics of flame HRR. However, it is worth noting that the use of measurement methods in nonpremixed flames is still a topic of controversy. Since it is beyond the scope of this paper to elucidate the mechanism of CH* generation during the nonpremixed combustion of hydrocarbon fuels, we mainly used the CH* signal as an indicator in this study to visualize the fluctuating trend of heat release rate. It is important to emphasize that while CH* signals were taken into account in our analysis, they were not the main focus of our investigation.

### 2.3. Operating Conditions

Experiments were conducted under ambient temperature and pressure to investigate the thermoacoustic instability of diffusion flames across different equivalence ratios. The inlet air temperature is 295 K, and the average pressure in the combustion chamber is 98 kPa. Throughout the experimental procedure, the mass flow rate of air, ${\dot{m}}_{\mathrm{air}}$, was maintained at a constant value of 5.4 g/s using a Mass Flow Controller (MFC). This configuration resulted in an axial velocity, u=17.8 m/s, at the nozzle exit. The equivalence ratio, ϕ, within the combustion chamber was adjusted by varying the fuel flow rate, ${\dot{m}}_{f}$. Table 1 presents the specific values of ${\dot{m}}_{f}$ and the corresponding ranges of ϕ for the tested conditions.

During the experiment, the ${\dot{m}}_{\mathrm{air}}$ was kept constant, and the ϕ in the combustion chamber was adjusted by regulating the C_3_H_8_ flow rate through an MFC. The control increment for ${\dot{m}}_{f}$ was set at 0.1 SLPM. Following each incremental adjustment, a stabilization period of approximately 10 min was observed to ensure steady-state operation conditions before commencing the recording of experimental data.

## 3. Results and Discussion

### 3.1. Stabilities Map and Thermoacoustic Modes

It is crucial to determine whether combustion is in a stable or unstable state. This article considers the criteria for combustion stability from two aspects: the amplitude of pressure fluctuations in the combustion chamber and the waveform of pressure fluctuations. On the one hand, the operating condition with the minimum root mean square pressure fluctuation, $P_{\mathrm{rms}}$, from different equivalence ratio test results is taken as the reference condition, $P_{\mathrm{rms},0}$. A dimensionless RMS value of pressure fluctuations is defined as

(3) $$\bar{P}=\frac{P_{\mathrm{rms}}}{P_{\mathrm{rms},0}}.$$

When $\bar{P}>3$, the combustion is deemed to be in an unstable state. On the other hand, the closer the combustion instability approaches a limit cycle state, the more the waveform of $p^{\prime }$ resembles a sinusoidal signal [30,31,32]. Conversely, the closer the combustion is to a stable state, the more the pressure waveform resembles the broadband random noise characteristic of combustion noise [33]. Based on previous studies on the kurtosis characteristics of $p^{\prime }$ [32], this paper defines a dimensionless kurtosis $\bar{K}$ to evaluate combustion stability:

(4) $$\bar{K}=\frac{K-1.5}{K_{R}-1.5}=\left(\frac{\sum_{i=1}^{n}{\left(p^{\prime }i-\bar{p}\right)}^{4}}{\sum {i=1}^{n}{\left(p_{i}^{\prime }-\bar{p}\right)}^{2}}-1.5\right)/1.5.$$

Here, *K* represents the kurtosis of the pressure oscillation $p^{\prime }$, and $\bar{p}$ is the mean value of $p^{\prime }$. For normally distributed random signals, K≈3, while for single-frequency modulated signals, K=1.5. Therefore, $\bar{K}$ reflects the degree to which the pressure oscillation signal deviates from a single-frequency sinusoidal signal. The higher the $\bar{K}$, the closer the signal is to a normally distributed random signal; conversely, the lower the $\bar{K}$, the closer $p^{\prime }$ is to a sinusoidal signal, indicative of a limit cycle state. In this study, $\bar{K}=0.6$ is chosen as the criterion for combustion instability. It should be emphasized that combustion is only considered unstable if the measured pressure oscillation results simultaneously meet both criteria.

**Figure 4:**
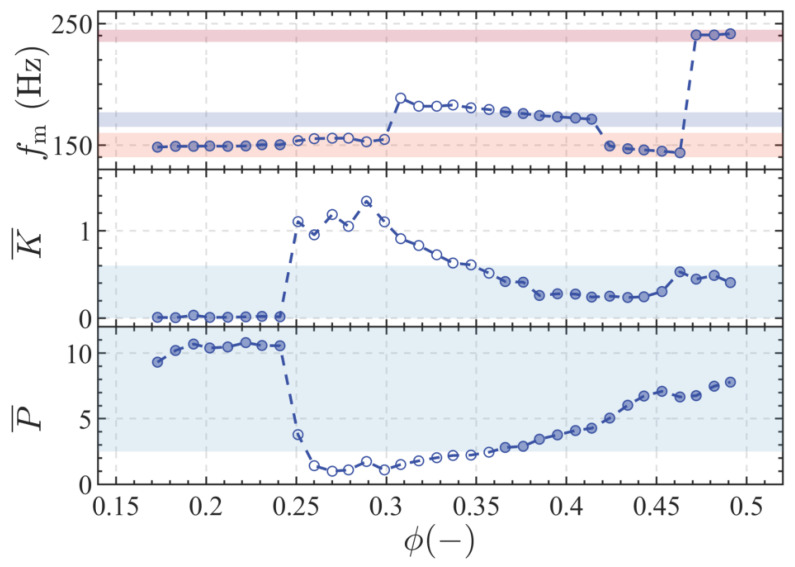
The stability map of the combustion chamber: $f_{m}$ (top), $\bar{K}$ (middle), and $\bar{P}$ (bottom) variation with equivalence ratio; the solid circles represent unstable conditions; the hollow circles represent stable conditions; and the shaded area indicates the range of critical $\bar{P}$ and $\bar{K}$ values.

Based on these criteria, the flame thermoacoustic stability boundaries at different inlet velocities are shown in Figure 4. At ϕ=0.173, the flame is in an unstable state, with $\bar{P}=7.64$ and $\bar{K}=0.009$. The dominant frequency, $f_{m}$, of pressure oscillations obtained through Fast Fourier Transform (FFT) is 150 Hz. As ϕ increases, more fuel is added to the combustion chamber, raising the combustion power and gas temperature and, thus, increasing the speed of sound and shifting the unstable frequency [19,34]. At ϕ=0.222, $f_{m}=150.3$ Hz, $\bar{P}$ and $\bar{K}$ are 10.8 and 0.010, respectively, reaching their maximum values. When ϕ reaches 0.260, combustion transitions to a stable state, with $\bar{P}$ dropping sharply from 10.56 to 3.79 and $\bar{K}$ increasing from 0.015 to 1.103.

As ϕ continues to increase, the flame transitions back into an unstable combustion state. At ϕ=0.385, $\bar{P}=3.44$, $\bar{K}=0.259$, and $f_{m}=174.3$ Hz. From these parameters, it can be seen that this unstable state differs from the state where $f_{m}$ is around 150 Hz, not only in terms of the dominant frequency but also in the lower amplitude of pressure oscillations and greater deviation from a single-frequency sinusoidal signal.

When ϕ increases to 0.424, thermoacoustic instability returns to the M1 state, with the main frequency of pressure oscillations being around 150 Hz. Compared to low-frequency instability at low equivalence ratios, the combustion chamber now exhibits a lower $\bar{P}$ and a higher $\bar{K}$. With further increases in equivalence ratio, when ϕ=0.472, the thermoacoustic instability has $\bar{P}=6.76$ and $\bar{K}=0.449$. This indicates that, compared to the previous state, the combustion chamber experiences greater pressure oscillations and more complex oscillatory waveforms under this unstable condition.

According to the experimental findings, for clarity and convenience, this study classifies different modes based on the frequency of thermoacoustic instability within the combustion chamber, labeling them as M1, M2, M11, and M3. Table 2 provides a list of representative operating conditions selected from each mode, which will serve as the primary subjects for subsequent analysis and discussion.

**Figure 5:**
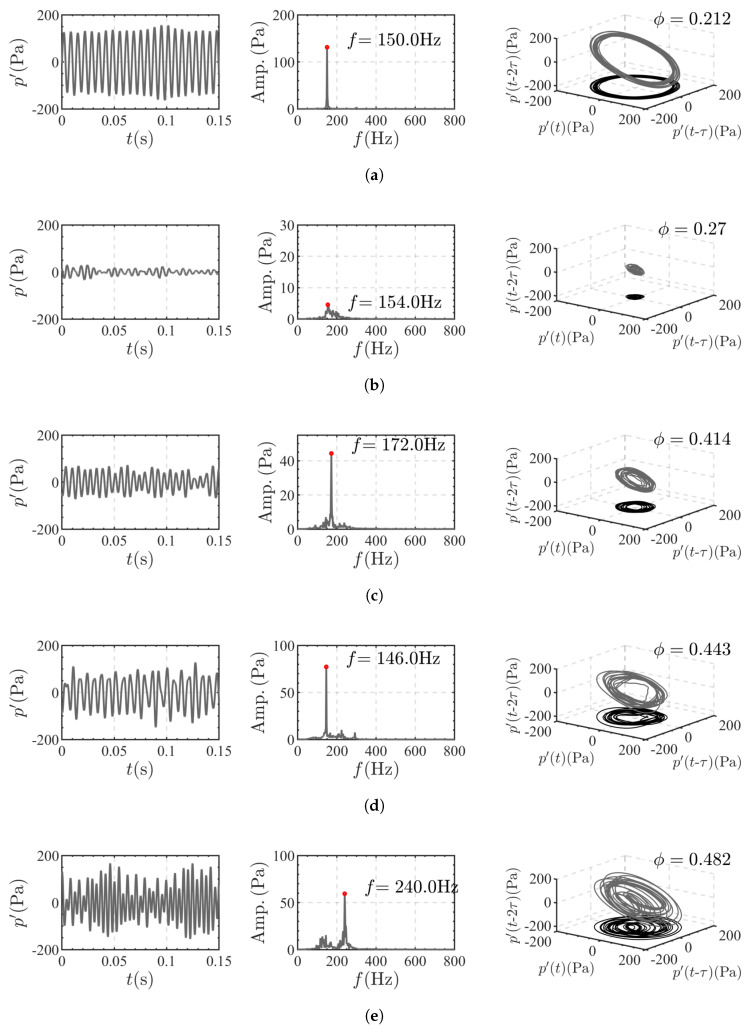
The pressure signal (left), frequency spectrum (middle), and phase space reconstruction (right) vary with ϕ: (**a**) ϕ = 0.212 (M1). (**b**) ϕ = 0.27 (MS). (**c**) ϕ = 0.443 (M2). (**d**) ϕ = 0.482 (M3). (**e**) ϕ = 0.482 (M3).

Figure 5 depicts the time-series pressure oscillation signals, FFT amplitude spectra, and limit cycle development through phase space reconstruction [35] under various equivalence ratio conditions. At ϕ=0.212, the pressure oscillation waveform is highly regular, with $\bar{K}$ significantly below 0.6, resembling a nearly pure sinusoidal signal. The dominant frequency amplitude is pronounced and stands out in the spectrum. The limit cycle is well-formed and characterized by a narrow ring. At ϕ=0.27, combustion is in a stable state. The amplitude of $p^{\prime }$ oscillations is low, and the dominant frequency is not prominent in the FFT spectrum. The phase space trajectory converges into a circular shape. Notably, this condition also serves as the reference case where $\bar{P}=1$. At ϕ=0.443, the system returns to the M1 state of thermoacoustic instability, with an increased amplitude of pressure oscillations compared to the M2 state. In the M3 state, compared to the M11 state, the amplitude of pressure oscillations in the spectrum is slightly reduced. The pressure oscillation waveform is more complex, and the phase space trajectory displays a dual-center structure. This indicates that the thermoacoustic instability in this state is approaching beat phenomena, explaining the higher $\bar{K}$ value observed in the M3 state.

### 3.2. Global Heat Release Rates Responses of Flames

**Figure 6:**
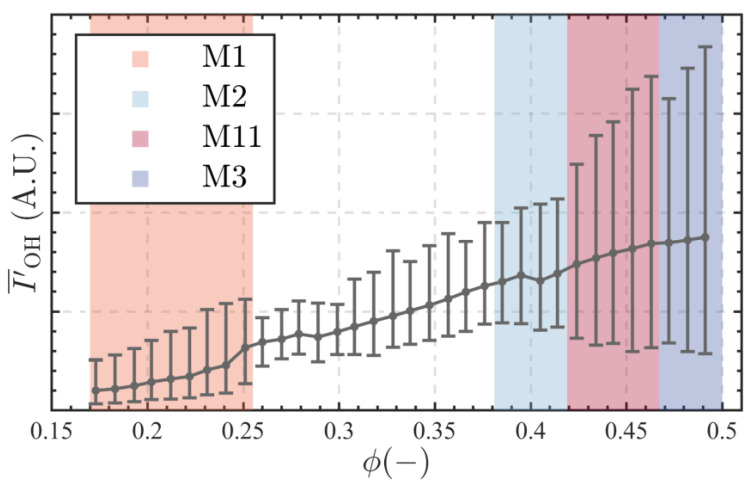
Global HRR trends characterized by OH emission intensity with respect to equivalence ratio ϕ.

To compare the characteristics of flame HRRs across different thermoacoustic instability modes, we first analyzed the global HRR oscillations at various equivalence ratios. Figure 6 shows the trends of global HRR, represented by OH* fluorescence intensity, as a function of ϕ. The data are normalized to the average luminous intensity at ϕ=0.173. As ϕ increases, the rise in combustion power leads to an increase in the average HRR.

- M1 State: The HRR oscillations are highly pronounced, with the peak-to-peak amplitude of $I_{\mathrm{OH}}^{\prime }$ being nearly twice the average value of ${\bar{I^{\prime }}}_{\mathrm{OH}}$. Additionally, the minimum value of $I_{\mathrm{OH}}^{\prime }$ remains relatively constant across different conditions, indicating the minimal level required to maintain stable ignition.
- M2 State: The difference between the maximum and minimum values of the HRR oscillations is comparable to the average HRR level. In this state, the flame exhibits relative stability, and the minimum value of the HRR oscillations gradually increases with increasing equivalence ratio, suggesting that the flame surface area remains largely unchanged.
- M11 State: The HRR oscillations are even more pronounced, characterized by an increase in the maximum value and a decrease in the minimum value of $I_{\mathrm{OH}}^{\prime }$. This suggests significant fluctuations in the heat release process, indicative of greater instability.
- M3 State: Compared to the M11 state, the HRR of the flame is slightly lower. However, the oscillatory behavior remains complex, reflecting the intricate dynamics of the system under this unstable condition.

### 3.3. Flame Dynamics of Different Modes

**Figure 7:**
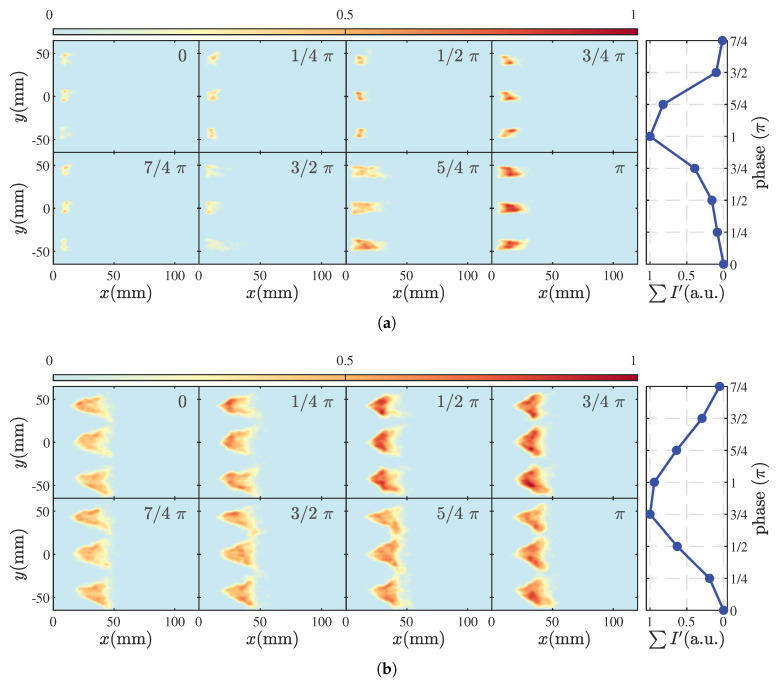
Phase-averaged flame dynamics in M1 (**a**) and M2 (**b**).

The fluctuation of the global OH HRR depicted in Figure 6 suggests the existence of differences in flame shapes across various states. Consequently, this paper presents phase-averaged results for different unstable states in Figure 7.

The phase-averaged outcomes were collected over 10 fluctuation cycles during the instability process. Each sequence is normalized by the maximum intensity in the cycle, and the last column of each row provides the normalized HRR level within one cycle. In state M1, shown in Figure 7a, at T=0, the flame attaches to the fuel injector exit with a relatively short length and is at its lowest HRR. As the phase advances, the flame gets gradually stretched, leading to an increase in flame length. When T=π, the HRR reaches its peak value. At T=5/4π, there is minimal change in flame length compared to the previous moment. However, due to fuel consumption, the overall HRR intensity of the flame slightly decreases. By T=3/2π, the fuel at the flame front is almost depleted, leading to pinch-off at the necking position, causing the flame front to exhibit irregular fragmentation due to breakage. At T=7/4π, the separated reactant pocket gradually extinguishes, and the flame shape returns close to the initial state at T=0, initiating the next cycle. This phenomenon aligns with the oscillation pattern of the global HRR shown in Figure 6. Similar processes dominated by fluctuations in flame length have been observed in premixed flames [36], partially premixed flames [13], and diffusion flames [19,22]. Under the M1 condition, high-amplitude pressure oscillations induce hydrodynamic disturbances that stretch and eventually separate the flame. Experiments [37] and numerical simulations [38] have confirmed that acoustic pressure perturbations can cause significant hydrodynamic oscillations. From a flame dynamics perspective, the phase-averaged results offer an explanation for the pronounced oscillation in the global HRR observed in state M1, as depicted in Figure 6.

In contrast, the M2 state, shown in Figure 7b, exhibits significant differences in flame morphology compared to the M1 state. Overall, these differences manifest in two aspects: First, with the increase in the equivalence ratio and the addition of more fuel, the airflow velocity at the injector exit increases, causing the flame to transition from an anchored state to a lifted state, and the flame shape changes from elongated to V-shaped. The topological transitions in nonpremixed flames have been extensively detailed in Ref. [39]. Second, from the perspective of flame geometry, there is no significant change in flame length. The flame structure mainly oscillates radially, whereas, in the M1 state, the flame oscillates axially. At T=0, the bottom of the flame has slight wrinkles, forming a tulip shape due to the swirling flames from the three injectors. At T=π, the HRR at the bottom of the flame initially strengthens, with the most noticeable change observed for the middle injector. By T=1/2π, the flame opening angle widens, and the HRR in the wrinkled area further increases. As *T* increases further, this wrinkle gradually propagates downstream and reaches its maximum value at T=π. With the increase in inlet air velocity, the height of the flame surface wrinkles gradually increases. This mode of flame fluctuation, characterized by expansion and contraction, leads to variations in flame surface area, resulting in the global HRR fluctuations shown in Figure 6. Under the influence of acoustic pressure fluctuations, the central recirculation zone of the swirling flame experiences periodic velocity fluctuations, and periodic sound-induced vortex structures appear in the inner and outer shear layers [40]. Their periodic generation, development, and dissipation are the primary reasons for the significant changes in the HRR.

**Figure 8:**
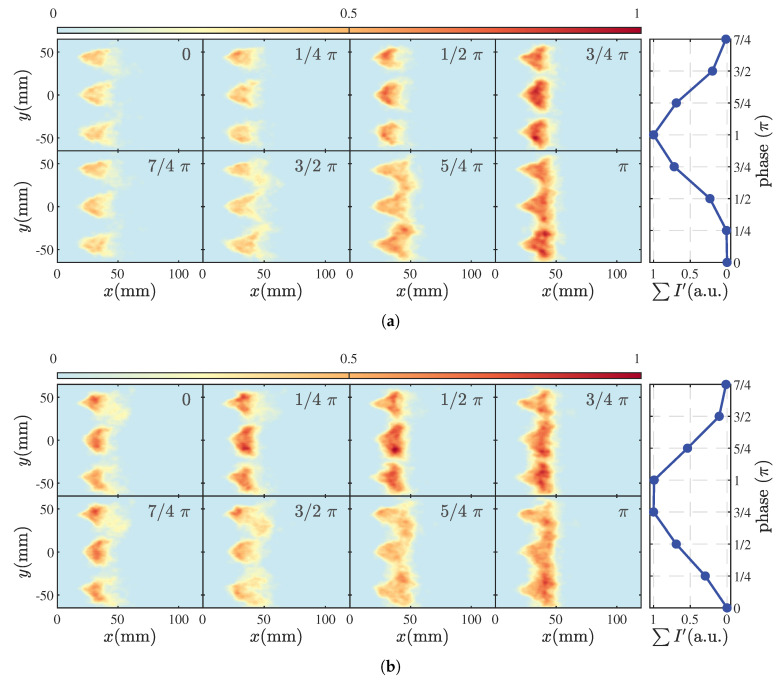
Phase-averaged flame dynamics in M11 (**a**) and M3 (**b**).

As the equivalence ratio increases further, the flame gradually transitions to the M11 state, as shown in Figure 8a, where it remains in a V-shaped lifted position similar to the M1 state. In this state, strong pressure oscillations within the combustion chamber lead to significant changes in flame length. Specifically, starting from T=0, the flame is progressively stretched and elongated, reaching its maximum length at T=π, when the HRR intensity at the flame front also peaks. In the subsequent development, the flame exhibits a fragmentation process similar to that in the M1 state and begins the next cycle of oscillation. Additionally, in the M11 state, there is a notable overlap at the flame front between T=π and T=5/4π. This overlap is even more pronounced in the M3 state.

Compared to the M1 and M2 states, the scenario depicted in Figure 8b displays more complex dynamic characteristics. The flame not only exhibits a curling, growth, and fragmentation process of the flame edge wrinkles similar to the M2 state but also shows flame breakage phenomena akin to those observed in the M11 state at T=3/2π and T=7/4π. These observations align with the pressure oscillation characteristics of the M3 state.

To further elucidate the spatiotemporal response differences in the HRR of flames under M1 and M2 modes, this study employed Proper Orthogonal Decomposition (POD) on CH luminescence images. The POD method extracts the spatial modes of flame fluctuations and their temporal distributions and is widely used in flame dynamics studies [16,17,19,20]. The POD algorithm used in this study was validated with data from [41].

**Figure 9:**
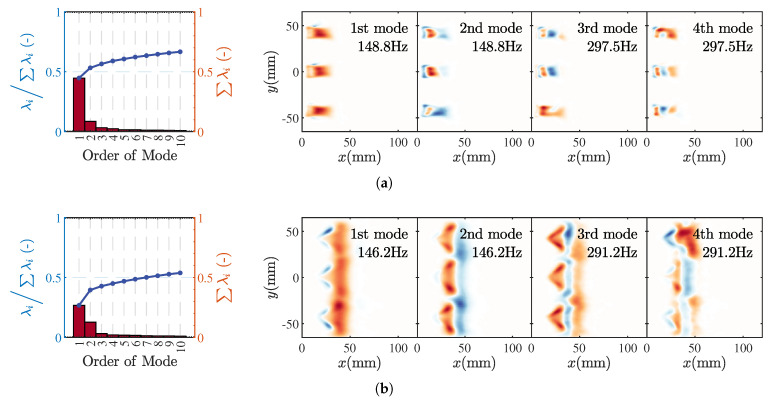
PODresults of CH luminescence images for flames in M1 (**a**) and M11 (**b**).

**Figure 10:**
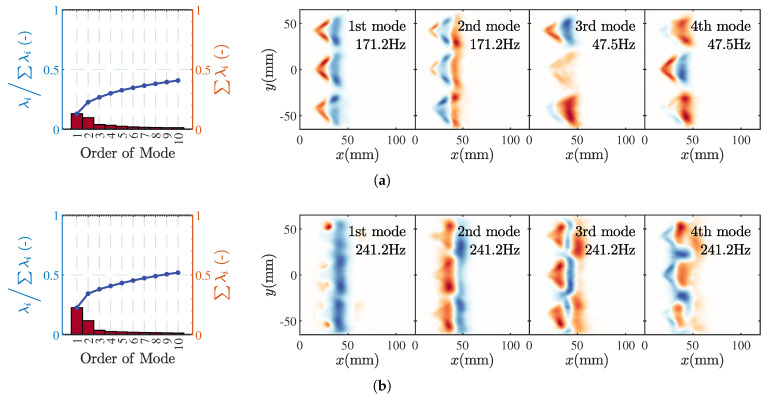
POD results of CH luminescence images for flames in M2 (**a**) and M3 (**b**).

Figure 9 and Figure 10 present the Proper Orthogonal Decomposition (POD) results of the flame CH* luminosity images under inlet velocity conditions corresponding to states M1 and M11 and M2 and M3, respectively. For the M1 state, the first-order mode of flame HRR fluctuation accounts for nearly half of the total fluctuation energy, with the top 10 modes collectively contributing approximately 0.7 of the total energy. The spatial distribution of the first mode covers the entire flame. According to the phase-averaged results of the flame shown in Figure 7, within one cycle, the flame sweeps from left to right, resulting in high fluctuation intensity. In the M1 state, the fluctuation frequency of the first-order mode is identical to the peak frequency of pressure fluctuations, indicating a coupling between pressure oscillations and the dominant mode of HRR fluctuations. The second-order mode results reveal longitudinal coherent structures within the flame, with opposite phases appearing at the necking position. Higher-order mode energies are relatively low and do not induce significant flame-stretching motions; these modes may originate from turbulence and turbulent combustion. In the M11 state, the energy proportion of the first-order mode decreases somewhat, with the top 10 modes accounting for about 0.52 of the total energy. The flame fluctuation structure exhibits typical longitudinal characteristics, where the V-shaped lifted flames formed by three injectors behave as a unified fluctuating structure. Additionally, in the fourth-order mode, the first injector displays an opposite spatial fluctuation structure.

Compared to the POD results in Figure 9, the M2 and M3 states exhibit notable differences. In terms of energy distribution, the first two fluctuation modes of the M2 flame have comparable energy levels, each accounting for approximately 0.15. The total energy contribution from the top 10 modes is about 0.4, which is significantly lower than the 0.7 observed in the M1 state. For the M2 state, in the first mode, oscillating bands of positive and negative amplitudes can be seen along the flame brush. The flame fluctuation structure is more concentrated near the edges of the V-shaped flame. This coherent structure corresponds to the flame pulsation pattern depicted in Figure 8, where the curling up and dissipation of flame edge wrinkles dominate the periodic HRR fluctuations. An intriguing observation is that in the third and fourth modes, the V-shaped flame formed by the central injector exhibits opposite spatial fluctuation structures compared to the upper and lower injectors, which could be one of the reasons for the relatively low amplitude of pressure fluctuations in the M2 state. In the POD results for the M3 state flame, both the proportion of the first mode and the total energy contribution of the top 10 modes are slightly increased. At this point, there is a more pronounced coupling between the flames produced by the three injectors. In the first mode, the V-shaped structure at the base of the flame is not as distinct; instead, it shows a longitudinal pulsation pattern involving the coupling of the three flames.

### 3.4. Phase-Space Distribution and Rayleigh Index Maps

**Figure 11:**
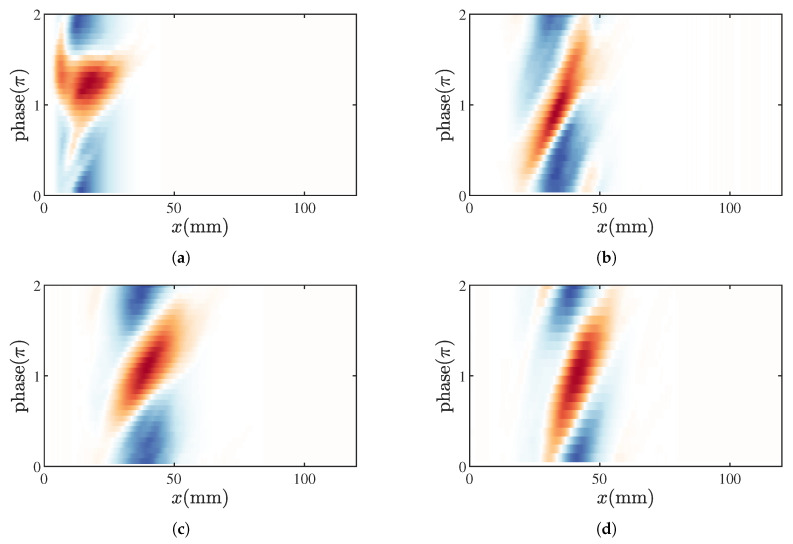
Phase-space distribution of HRR oscillations for M1 (**a**), M2 (**a**), M11 (**c**), and M3 (**d**).

To more intuitively analyze the differences in flame HRR responses under two modes, Figure 11 provides phase-space diagrams [10,42] of the flame HRR for different modes. In these phase-space diagrams, the horizontal axis represents the axial co-ordinate of the flame, while the vertical axis indicates the phase. The contours reflect changes in the flame HRR relative to the time-averaged results. Under the M1 and M11 modes, the phase-space distribution of the flame HRR is essentially identical, with differences only appearing in the flame distribution areas affected by changes in flame shape. Within one cycle, the flame undergoes stretching, separation, and fracture processes. In contrast, the higher instability frequencies in the M2 and M3 states result in more fluctuation bands within a single cycle. The slope of these bands is also significantly steeper than that of the M1 and M11 states. The flame HRR is primarily concentrated at the flame front. In the phase-space results, the slope of the bands satisfies the following:

(5) $$\frac{d\varphi }{dx}=2\pi f\frac{dt}{dx}=2\pi fu$$

where *f* is the fluctuation frequency, and *u* is the convective velocity. In the M2 and M3 states, the greater slope of the bands indicates a higher convective velocity, corresponding to the curling up of flame wrinkles and their downstream convective transport. Specifically, in the M2 state, as the inlet velocity increases, the flame bands narrow, indicating a shorter convective wavelength.

Based on the discussion of the spatiotemporal fluctuation characteristics of the flame HRR, it has been noted that there are significant differences in the HRR response during thermoacoustic instability between different instability states. Therefore, this study further investigates the spatial distribution of the Rayleigh Index (RI). The RI is defined as

(6) $$RI\left(x,y\right)=\frac{1}{T}\int_{0}^{T}q^{\prime }\left(x,y\right)p^{\prime }\left(x,y\right)dt.$$

**Figure 12:**
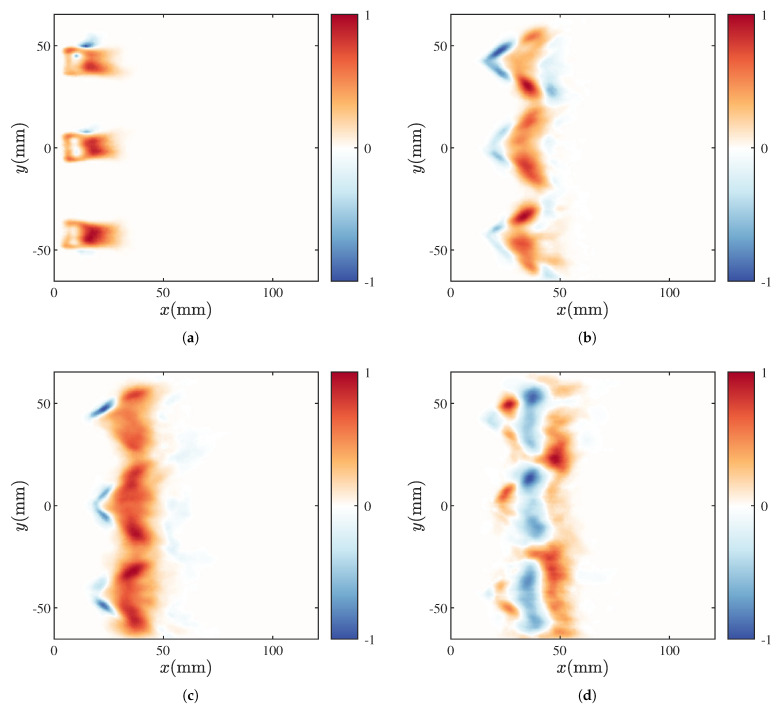
Spatial distribution of the Rayleigh Index for the M1 (**a**), M2 (**a**), M11 (**c**), and M3 (**d**) states.

Given that the flame length is much shorter compared to the characteristic wavelength within the combustion chamber, $p^{\prime }$ is used instead of $p^{\prime }\left(x,y\right)$ for the calculation. Figure 12 provides the RI distributions for the M1 and M2 states. Under M1 and M11 conditions, almost the entire flame contributes to driving the thermoacoustic instability, with the strongest driving region located downstream of the flame brush; damping effects are only observed over a very small range in the middle of the flame. In contrast, under M2 and M3 conditions, regions of damping and gain alternate across the flame surface, with the largest gain occurring at the flame front. This alternating effect of flame driving and damping also explains the lower amplitude of pressure oscillations observed in the M2 and M3 modes. These results further indicate that, compared to the M2 state, the gain regions in the M3 state are primarily located at the flame front, whereas in the M2 state, they are mainly concentrated in the middle section of the flame. Additionally, the base of the V-shaped flame exhibits pronounced damping characteristics.

During a fluctuation cycle, an increase in combustion chamber pressure implies a reduction in air velocity at the injector outlet. The flame base propagates upstream along the stoichiometric isosurface, resulting in an increase in HRR. When the combustion chamber pressure decreases, the air velocity at the injector outlet increases, which hinders the flame’s ability to anchor at its original position, causing it to move downstream and reducing the HRR near the injector tip.

## 4. Conclusions

This study has provided a comprehensive analysis of the flame dynamics in a triple-injector swirl nonpremixed combustor under various thermoacoustic self-excited instability modes. The experimental approach, utilizing dynamic pressure sensors and high-speed imaging, allowed for detailed observations of the spatiotemporal characteristics of flame HRR fluctuations. The modes of thermoacoustic instability and the HRR response have been analyzed under various equivalence ratios. The main conclusions of this study are summarized as follows:

In this study, dynamic pressure sensors and high-speed imaging techniques were employed to capture pressure oscillations within the combustion chamber and the characteristics of flame dynamics, respectively. The application of these sensors not only provided critical information for identifying different thermoacoustic instability modes but also helped in understanding the complex interactions between flame behavior and thermoacoustic instabilities more accurately.

When the air flow rate in the combustion chamber is kept constant, the chamber exhibits nonlinear bifurcation behavior as the equivalence ratio increases. Different self-excited thermoacoustic instability modes have distinct pressure pulsation characteristics and global HRR pulsation characteristics, as discussed in Section 3.1 and Section 3.2. The flame dynamics differ significantly across these modes. In the M1 and M11 modes, the pulsation of the flame length plays a crucial role in driving the thermoacoustic instability in low-frequency modes. In the M2 mode, the HRR pulsation characteristics are dominated by convectively transported acoustic-induced vortex modes. Flame pinch-off dynamics and the rolling up and convective transport of flame wrinkles are observed simultaneously in the M3 mode, with detailed results presented in Section 3.3 and Section 3.4. These results offer valuable insights into the complex interactions between flame dynamics and thermoacoustic instabilities, which are crucial for the design and optimization of nonpremixed combustion systems. The study emphasizes the importance of considering the spatial and temporal variations in flame behavior to effectively predict and control thermoacoustic instabilities.

Through the analysis of sensor data under varying equivalence ratio conditions, we found that the temporal and spatial variations in flame behavior are crucial for predicting and controlling thermoacoustic instability. Particularly in multi-nozzle layout combustors, where complex interactions between nozzles must be considered, the detailed spatiotemporal resolution data provided by sensors are essential. These data not only help explain the observed phenomena but also offer new perspectives for optimizing the stability and performance of nonpremixed combustion systems.

Future research could further explore how advanced sensing technologies can be utilized for real-time monitoring and control of thermoacoustic instability during combustion processes. This includes developing sensors with higher sensitivity and resolution and integrating machine learning algorithms to automatically analyze large datasets from sensors, enabling precise prediction and effective control of combustion instabilities.

In summary, by thoroughly analyzing sensor data, this study has revealed the complex relationship between flame dynamics and thermoacoustic instability, providing valuable insights for the design and optimization of nonpremixed combustion systems. It also underscores the importance of considering the spatial and temporal variations in flame behavior in practical engineering applications to better predict and control thermoacoustic instability within combustion chambers.

## Figures and Tables

**Table 1:** Overview of operating conditions.

$T_{\mathrm{in}}$	$p_{c}$	${\dot{m}}_{\mathrm{air}}$	Range of ${\dot{m}}_{f}$	Range of ϕ
(K)	(Pa)	(g/s)	(SLPM)	(-)
295	98 k	5.4	1.8–5.1	0.173–0.491

**Table 2:** Representative operating conditions.

No	ϕ	$f_{m}$	$\bar{K}$	$\bar{P}$
	(-)	(Hz)	(-)	(-)
M1	0.212	149.0	0.010	10.47
MS *	0.270	155.7	1.184	1.0
M2	0.414	172.0	0.275	4.10
M11	0.443	146.0	0.244	6.73
M3	0.482	240.0	0.488	7.48

* MS stands for stable conditions.

## Data Availability

The data that support the findings of this study are available from the corresponding author upon reasonable request.
